# On-line memory clinic – piloting a hybrid model

**DOI:** 10.1192/bjo.2021.601

**Published:** 2021-06-18

**Authors:** Rahul Tomar

**Affiliations:** Logandene, Hertfordshire Partnership University NHS Foundation NHS Trust

## Abstract

**Aims:**

Quality improvement project was undertaken to reorganise memory clinic to incorporate both virtual and in-person consultation (Hybrid Virtual model), as depicted in the following model:

**Method:**

Tele triage conducted to ascertain information from patient and carer. This reduced time for face to face assessment.

Nurse did face to face assessment to complete cognitive test (Addenbrooke's Cognitive Examination III) & carer completed Bristol Activities of Daily Living scale. Nurse would also do BP, PR, oxygen sats & temp.

Nurse discussed the assessment with the consultant (who is in the inpatient unit) on line using MS TEAMS

Consultant would then see patient on line, confirm diagnosis, answer questions, give information on medication and post prescription (if required)

Feedback was collected using Telehealth Satisfaction questionnaire

**Result:**

Hybrid remote memory clinic was started on 29/09/20. A total of 37 patients were seen in this clinic by 31/01/21.

Collected feedback from 21 patients was generally positive –

Information provided on video consultation prior to assessment -18 reported it as excellent

How well you privacy was respected – 21 reported it as excellent.

Information you received on the treatment – 18 reported it as excellent

**Conclusion:**

The hybrid remote memory clinic was more effective than telephone consultation or on line only consultation as it was –

Easy to establishing rapport

Physical examination could be performed

Digital literacy was no longer a limiting factor

Prescribing medication was slightly more difficult but possible

